# Novel Glycemic Index Based on Continuous Glucose Monitoring to Predict Poor Clinical Outcomes in Critically Ill Patients: A Pilot Study

**DOI:** 10.3389/fendo.2022.869451

**Published:** 2022-05-04

**Authors:** Eun Yeong Ha, Seung Min Chung, Il Rae Park, Yin Young Lee, Eun Young Choi, Jun Sung Moon

**Affiliations:** ^1^Division of Endocrinology and Metabolism, Department of Internal Medicine, College of Medicine, Yeungnam University, Daegu, South Korea; ^2^Division of Endocrinology and Metabolism, Department of Internal Medicine, Veterans Health Service Medical Center, Daegu, South Korea; ^3^Division of Pulmonology and Allergy, Department of Internal Medicine, Respiratory Center, Yeungnam University Medical Center, College of Medicine, Yeungnam University, Daegu, South Korea

**Keywords:** blood glucose, diabetes mellitus, glucose, hospitals, intensive care units, technology

## Abstract

**Aim:**

We explored the prospective relationship between continuous glucose monitoring (CGM) metrics and clinical outcomes in patients admitted to the intensive care unit (ICU).

**Materials and Methods:**

We enrolled critically ill patients admitted to the medical ICU. Patients with an Acute Physiology and Chronic Health Evaluation (APACHE) score ≤9 or ICU stay ≤48 h were excluded. CGM was performed for five days, and standardized CGM metrics were analyzed. The duration of ICU stay and 28-day mortality rate were evaluated as outcomes.

**Results:**

A total of 36 patients were included in this study (age [range], 49–88 years; men, 55.6%). The average APACHE score was 25.4 ± 8.3; 33 (91.7%) patients required ventilator support, and 16 (44.4%) patients had diabetes. The duration of ICU stay showed a positive correlation with the average blood glucose level, glucose management indicator (GMI), time above range, and GMI minus (-) glycated hemoglobin (HbA1c). Eight (22.2%) patients died within 28 days, and their average blood glucose levels, GMI, and GMI-HbA1c were significantly higher than those of survivors (p<0.05). After adjustments for age, sex, presence of diabetes, APACHE score, and dose of steroid administered, the GMI-HbA1c was associated with the risk of longer ICU stay (coefficient=2.34, 95% CI 0.54-4.14, p=0.017) and higher 28-day mortality rate (HR=2.42, 95% CI 1.01-5.76, p=0.046).

**Conclusion:**

The acute glycemic gap, assessed as GMI-HbA1c, is an independent risk factor for longer ICU stay and 28-day mortality rate. In the ICU setting, CGM of critically ill patients might be beneficial, irrespective of the presence of diabetes.

## Introduction

Acute hyperglycemia is commonly encountered in critically ill patients admitted to the intensive care unit (ICU), regardless of the presence of diabetes mellitus (DM) (1). Hyperglycemia is induced by acute stress and is also associated with the prognosis of severely ill patients (2). In addition, these patients are vulnerable to hypoglycemia, both iatrogenic and idiopathic, and several studies have suggested that hypoglycemia is an independent risk factor for mortality. Recent guidelines recommend that the goal of glycemic control in the ICU is 140–180 mg/dL, although there are controversies about the appropriate target range. These findings emphasize the importance of glucose monitoring and management in critically ill patients. However, point-of-care (POC) blood glucose monitoring has limitations in ICU settings, such as missing or not being able to predict hypoglycemia or hyperglycemia. In addition, though glycated hemoglobin (HbA1c) is an important indicator of the condition of diabetic patients and the risk of long-term diabetic complications (3), this may be insufficient to optimally induce personalized treatment changes, especially in patients using insulin, as the degree or timing of hypoglycemia, and the presence of clinically significant glucose variability or hyperglycemic patterns are unknown. Previous studies have demonstrated glucose variability with mean amplitude of glucose excursion (MAGE), continuous overall net glycemic action (CONGA), and M-values (4–6). However, these require the use of a special calculation program and are difficult to calculate and apply immediately in ICU patients. On the other hand, continuous glucose monitoring (CGM) is a great help in evaluating blood glucose variability as it can easily obtain sufficient data (7).

CGM is a powerful tool with the potential to transform the management of individuals with diabetes. In real time, CGM can show trends in hypoglycemia, hyperglycemia, and glucose variability, some of which warrant immediate therapeutic action (8, 9). In other words, CGM helps individuals with diabetes and clinicians optimize diabetes management strategies. CGM is strongly recommended in clinical situations requiring intensive glucose monitoring, such as patients receiving multiple insulin injections (10–12). The benefits of CGM include the prediction and prevention of rapid glycemic changes, which cannot be recognized with POC, HbA1c, glycated albumin, or fructosamine, and this technology will be accepted in various situations including in-hospital care (13, 14).

CGM has also been highlighted as an attractive alternative to hourly POC in the ICU and shows high accuracy and reliability in patients admitted to cardiac, surgical, and medical ICUs, as well as patients with coronavirus disease 2019 (15–18). The use of CGM metrics for remote blood glucose monitoring in the ICU has been approved due to the recent coronavirus disease 2019 pandemic (19, 20). However, little is known about the clinical usefulness or implications of CGM metrics in ICU settings. Therefore, we aimed to investigate the correlation of CGM metrics with clinical outcomes in critically ill patients admitted to the medical ICU. In addition, we attempted to devise a novel index based on conventional CGM metrics to predict the prognosis of patients admitted to the ICU.

## Materials and Methods

### Study Design and Patient Selection

This prospective observational study enrolled critically ill patients admitted to the medical ICU of Yeungnam University Hospital, Daegu, South Korea, between June 2020 and February 2021. The study was conducted after the patient or legal representative provided written informed consent. We initially selected 52 patients and examined their eligibility. The inclusion criteria were as follows: 1) patients aged >45 years and 2) critically ill patients who were admitted due to pneumonia, septic shock, or acute respiratory distress syndrome (ARDS). The exclusion criteria were as follows: 1) patients whose expected the duration of ICU stay was ≤48 h, 2) patients with an Acute Physiology and Chronic Health Evaluation (APACHE) II score ≤9, 3) patients with chronic disease who were less likely to be resuscitated, and 4) patients with a high risk of bleeding during CGM (platelet count < 50,000/µL). A total of 36 patients were included in the final analysis. The study protocol adhered to the tenets of the Declaration of Helsinki and was reviewed and approved by the Institutional Review Board of Yeungnam University Hospital (approval no. 2019-07-043).

### Clinical and Biochemical Measurements

Disease severity was assessed using the APACHE II score (21). Higher scores (range, 0–71) are closely correlated with the subsequent risk of in-hospital death: an APACHE II score ≥10 reflects an estimated in-hospital mortality of >15%. ARDS was diagnosed according to the Berlin definition (22). Septic shock was defined according to the Third International Consensus Definitions for Sepsis and Septic Shock (Sepsis-3) (23). Data on ventilator support and administration of steroids or insulin during ICU care were collected.

Waist circumference and blood pressure were measured by trained staff members. All laboratory parameters were evaluated at the central laboratory of Yeungnam University Hospital. The white blood cell and platelet counts, and hemoglobin (Hb), C-reactive protein (CRP), procalcitonin, glycated hemoglobin (HbA1c), fasting glucose, fasting insulin, total cholesterol, triglyceride, high-density lipoprotein cholesterol, and creatinine levels were measured.

The diagnostic criteria for DM were as follows: 1) previous diagnosis by a doctor or 2) satisfying the following conditions: fasting plasma glucose ≥ 126 mg/dL and HbA1c ≥ 6.5% (24).

### CGM and Initiation of Insulin Infusion

For glucose monitoring, a CGM system (Dexcom G5, Dexcom, San Diego, USA) was attached for 5 days immediately after the admission, and calibration was performed using the same self-monitoring glucometers at least twice a day to increase the accuracy of the data. Venous blood glucose levels were checked to ensure that the CGM system was functioning properly. The transmitter was removed during radiography or computed tomography.

Irrespective of the presence of DM, patients with two or more instances of blood glucose levels > 180 mg/dL were initiated on the Yale ICU insulin infusion protocol (25). The target blood glucose range was 140–180 mg/dL. Based on the International Consensus statement, the following key CGM metrics were collected (26): average blood glucose level, glucose management indicator (GMI), coefficient of variation (CV), time in range (TIR, 70–180 mg/dL), time above range (TAR, > 180 mg/dL), and time below range (TBR, < 70 mg/dL). In addition, we analyzed the difference between GMI and HbA1c levels (GMI-HbA1c) (8). We also calculated other CGM-derived metrics such as MAGE, CONGA, and M-values using EasyGV software (www.phc.ox.ac.uk/research/resources/easygv).

### Outcomes

The outcomes evaluated were the duration of ICU stay and 28-day mortality rate. *Post-hoc* power was calculated using previously published data (27) as known population, and the *post-hoc* power of our study was 50.7%.

### Statistical Analysis

All statistical tests were performed using R software (version 3.6.3, R Foundation, Vienna, Austria). Baseline characteristics are expressed as mean ± standard deviation for continuous variables and as numbers and percentages for categorical variables. Differences between groups were assessed using the Mann–Whitney U test for continuous variables and chi-square tests for categorical variables. Spearman’s correlation analysis was used to assess the correlation between CGM metrics and duration of ICU stay. Linear regression analysis was used to assess the effects of CGM metrics on the duration of ICU stay. Cox regression analysis was used to assess the effects of CGM metrics on 28-day mortality rate. Hazard ratios (HRs) were reported with 95% confidence intervals (CIs). Statistical significance was set at P < 0.05.

## Results

### Baseline Characteristics

Baseline characteristics and their comparisons between patients with and without diabetes are presented in **Table 1**.

**Table 1:** Comparison of baseline characteristics between patients with and without diabetes.

	Overall (n=36)	non-DM (n=20)	DM (n=16)	p-value
Age, years	71.0 ± 9.9	69.5 ± 11.0	72.9 ± 8.1	0.305
Men, n (%)	20 (55.6)	11 (55.0)	9 (56.2)	
Diagnosis at ICU admission				
Pneumonia, n (%)	21 (58.3)	11 (55.0)	10 (37.0)	0.711
Septic shock, n (%)	8 (22.2)	4 (20.0)	4 (14.8)	
ARDS, n (%)	7 (19.4)	5 (25.0)	2 (7.4)	
Disease severity at ICU admission				
APACHE	25.4 ± 8.3	25.1 ± 8.8	25.8 ± 7.9	0.765
Laboratory data				
Waist circumference, cm	89.5 ± 11.2	89.3 ± 10.7	89.9 ± 12.2	0.959
Systolic BP, mmHg	118.7 ± 25.8	118.2 ± 27.1	119.4 ± 25.0	0.694
Diastolic BP, mmHg	86.8 ± 91.4	97.6 ± 121.7	73.2 ± 19.4	0.962
WBC, x10^9^/L	12.3 ± 5.8	12.3 ± 6.2	12.2 ± 5.5	0.814
Hb, g/dL	11.2 ± 1.9	11.5 ± 1.9	10.8 ± 2.0	0.237
Platelet, x10^9^/L	241.1 ± 93.9	252.2 ± 86.2	227.2 ± 103.9	0.626
CRP, mg/dL	16.9 ± 11.9	16.1 ± 12.3	18.0 ± 11.8	0.604
Procalcitonin, mg/dL	5.8 ± 12.9	4.9 ± 11.9	6.8 ± 14.4	0.249
HbA1c, %	6.8 ± 1.7	5.9 ± 0.5	8.0 ± 2.0	<0.001
Fasting glucose, mg/dL	194.2 ± 85.4	194.8 ± 85.2	193.4 ± 88.4	0.987
Fasting insulin, uIU/mL	37.1 ± 52.3	39.7 ± 55.4	33.9 ± 49.7	0.178
Total cholesterol, mg/dL	116.9 ± 53.8	128.4 ± 64.6	102.5 ± 32.8	0.305
Triglyceride, mg/dL	129.8 ± 82.8	138.1 ± 100.9	119.4 ± 53.8	0.838
HDL cholesterol, mg/dL	29.1 ± 13.7	32.1 ± 15.7	25.3 ± 10.0	0.149
Creatinine, mg/dL	1.4 ± 1.2	1.1 ± 0.8	1.8 ± 1.6	0.095
CGM metrics				
Days CGM worn, days	5.5 ± 0.8	5.5 ± 0.8	5.4 ± 0.8	0.369
Time CGM is Active, %	98.5 ± 3.1	98.4 ± 2.3	98.6 ± 3.9	0.479
Average glucose, mg/dL	162.2 ± 43.2	150.2 ± 40.6	177.2 ± 42.8	0.030
GMI, %	7.3 ± 1.5	6.9 ± 1.4	7.8 ± 1.5	0.033
CV, %	28.2 ± 9.1	25.1 ± 7.3	32.0 ± 9.9	0.028
TAR, %	29.8 ± 26.5	22.5 ± 25.9	38.8 ± 25.0	0.030
TIR, %	69.2 ± 26.2	76.5 ± 25.5	60.0 ± 24.8	0.039
TBR, %	1.1 ± 2.3	1.0 ± 2.2	1.2 ± 2.5	0.814
GMI-HbA1c, %	0.4 ± 1.7	1.0 ± 1.3	-0.2 ± 2.0	0.020
Treatment				
Ventilator care, n (%)	33 (91.7)	19 (95.0)	14 (87.5)	0.574
Steroid, n (%)	27 (75.0)	14 (70.0)	13 (81.2)	0.7
Insulin, n (%)	24 (66.7)	11 (55.0)	13 (81.2)	0.192
Outcome				
ICU stay, days	10.8 ± 7.6	10.2 ± 6.5	11.6 ± 8.9	0.789
28-day mortality rate, n (%)	8 (22.2)	5 (25.0)	3 (18.8)	0.709

APACHE, Acute Physiology and Chronic Health Evaluation; ARDS, acute respiratory distress syndrome; BP, blood pressure; CGM, continuous glucose monitoring; CRP, C-reactive protein; CV, coefficient of variation; DM, diabetes mellitus; GMI, glucose management indicator; HbA1c, glycated hemoglobin; HDL, high-density lipoprotein; ICU, intensive care unit; TAR, time above range; TBR, time below range; TIR, time in range; WBC, white blood cell.

The mean age was 71.0 ± 9.9 years (range, 49–88 years), and the male-to-female ratio was 1.25:1. The most common diagnosis at the time of admission was pneumonia (58.3%), followed by septic shock (22.2%) and ARDS (19.4%). The mean APACHE score was 25.4 ± 8.3. The CGM system was attached for 5.5 ± 0.8 days and activated for 98.5% ± 3.1% of time. Thirty-three patients (91.7%) required ventilator support. Steroids were administered to 75% of patients, and insulin was administered to 66.7% of patients. The average duration of ICU stay was 10.8 ± 7.6 days, and 8 patient (22.2%) died within 28 days.

Among all patients, 16 (44.4%) had DM. Thirteen patients were previously diagnosed by a doctor; however, 10 of them did not receive anti-hyperglycemic treatment. Three patients were newly diagnosed at the time of admission. Age, sex, diagnosis, and disease severity at the time of admission were not different between patients with and without DM. The white blood cell count and levels of CRP and procalcitonin were increased in patients with and without DM without statistically significant differences. The average HbA1c level of patients with DM was 8.0%, and that of patients without DM was 5.9% (p<0.001). Compared to patients with DM, the average blood glucose level (150 mg/dL vs. 177 mg/dL, p=0.030), GMI (6.9% vs. 7.8%, p=0.033), CV (25.1% vs. 32.0%, p=0.028), and TAR (22.5% vs. 38.8%, p=0.030) were significantly lower, and the TIR (76.5% vs. 60.0%, p=0.039) and GMI-HbA1c (1.0% vs. -0.2%, p=0.020) were significantly higher in non-diabetic patients. Treatment, including ventilator support, steroids, and insulin, and clinical outcomes (ICU stay and 28-day mortality rate) did not differ significantly between patients with and without DM.

### Association Between CGM Metrics and Duration of ICU Stay

**Figure 1:**
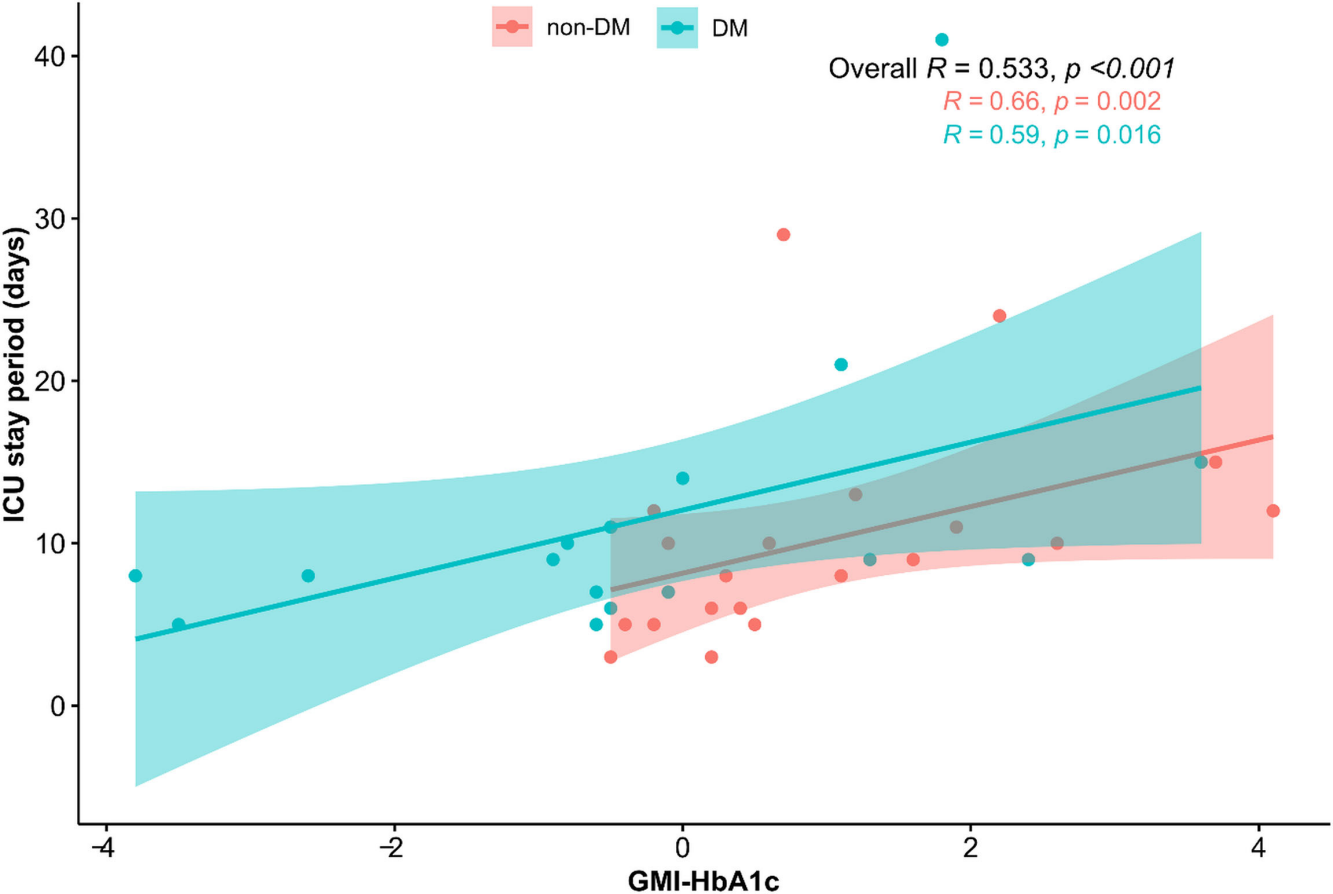
Scatter plot of the relationship between GMI-HbA1c and ICU stay. The correlation coefficients are presented as Spearman’s r.

The correlation analysis for CGM metrics and ICU stay is presented in **Table 2**. The average blood glucose level (r=0.532, p<0.001), GMI (r=0.545, p<0.001), TAR (r=0.457, p=0.005), and GMI-HbA1c (r=0.533, p<0.001) were positively associated, and TIR (r=-0.435, p=0.008) was negatively associated with the duration of ICU stay. GMI-HbA1c was positively correlated with duration of ICU stay in both patients without DM (r=0.66, p=0.002) and patients with DM (r=0.59, p=0.016; **Figure 1**). However, the correlation between duration of ICU stay and CV or TBR was not significant. Other CGM-derived parameters, such as MAGE, CONGA, and M-value, were also not correlated to ICU stay length (**Supplementary Table 1**).

**Table 2:** Correlation between CGM metrics and ICU stay.

	Correlation coefficients	p
Average glucose, mg/dL	0.532	<0.001
GMI, %	0.545	<0.001
CV, %	0.051	0.767
TAR, %	0.457	0.005
TIR, %	-0.435	0.008
TBR, %	-0.213	0.212
GMI-HbA1c, %	0.533	<0.001

The correlation coefficients are presented as Spearman r.

CV, coefficient of variation; DM, diabetes mellitus; GMI, glucose management indicator; HbA1c, glycated hemoglobin; TAR, time above range; TBR, time below range; TIR, time in range.

### Association Between CGM Metrics and 28-Day Mortality Rate

**Figure 2:**
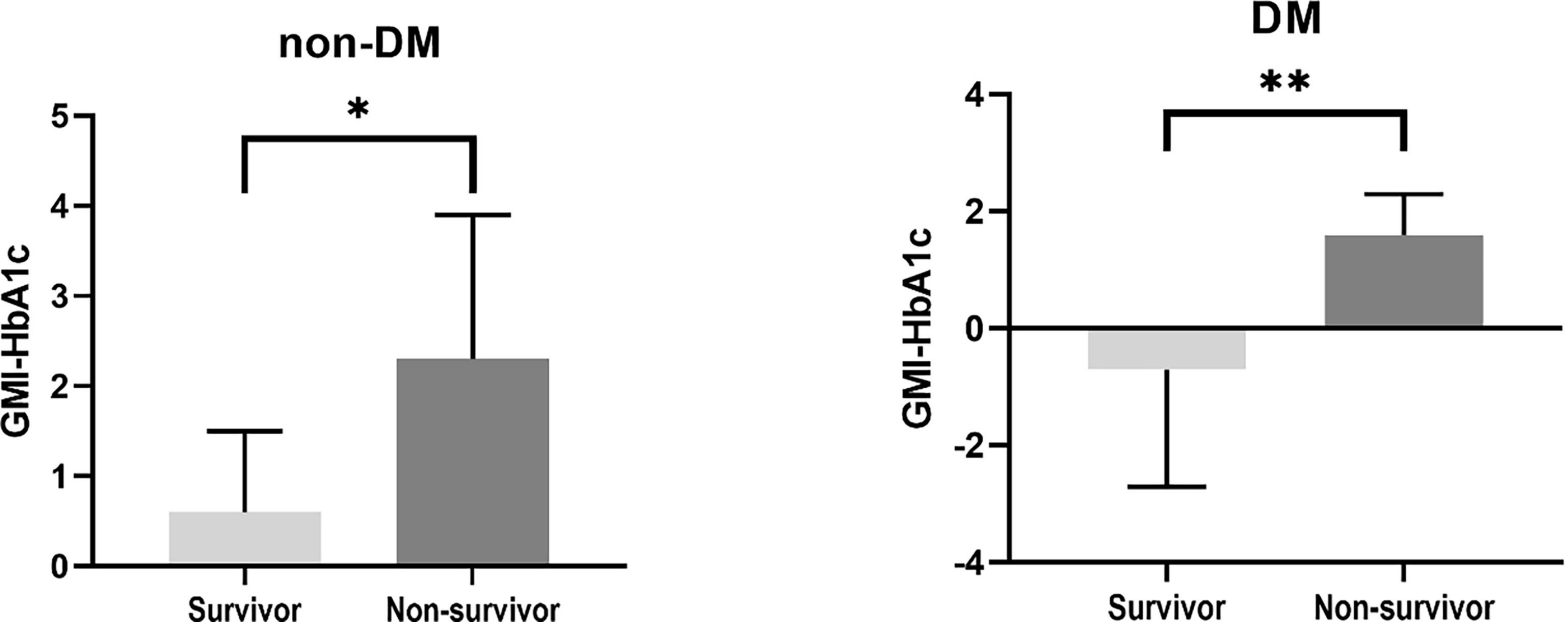
Difference in GMI-HbA1c between survivors and non-survivors. *p < 0.05, **p < 0.01.

Clinical characteristics of the survivors and non-survivors at 28 days are presented in **Table 3**. Eight patients died within 28 days. There were no differences in age, sex, presence of DM, and APACHE scores between survivors and non-survivors. In non-survivors, the prevalence of ARDS was significantly higher (75% vs. 3.6%, p=0.001), and a higher dose of steroid was administered (61.7 mg/day vs. 25.6 mg/day, p=0.007) than in survivors. Among CGM metrics, the average blood glucose level (188.8 ± 35.6 vs. 154.6 ± 42.7, p=0.021), GMI (8.2 ± 1.3 vs. 7.0 ± 1.5, p=0.024), and GMI-HbA1c (2.0 ± 1.3 vs. 0.0 ± 1.6, p<0.001) were significantly higher in non-survivors than in survivors. The GMI-HbA1c was significantly higher in non-survivors than in survivors in both patients without DM (2.3 ± 1.6 vs. 0.6 ± 0.9, p=0.025) and patients with DM (1.6 ± 0.7 vs. -0.7 ± 2.0, p=0.008; **Figure 2**). M-value was also marginally higher in the non-survivor group (p=0.059), but MAGE was not different between group (**Supplementary Table 1**).

**Table 3:** Comparison of characteristics according to mortality within 28 days.

	Survivor (n=28)	Non-survivor (n=8)	p-value
Age, years	70.9 ± 10.1	71.2 ± 9.7	0.537
Men, n(%)	16 (57.1)	4 (50.0)	1
DM, n(%)	13 (46.4)	3 (37.5)	0.709
Diagnosis at ICU admission			
Pneumonia, n (%)	20 (71.4)	1 (12.5)	0.001
Septic shock, n (%)	7 (25.0)	1 (12.5)	
ARDS, n (%)	1 (3.6)	6 (75.0)	
Disease severity at ICU admission			
APACHE	25.2 ± 8.5	26.2 ± 8.1	0.668
HbA1c, %	7.0 ± 1.9	6.2 ± 0.5	0.236
Fasting glucose, mg/dL	183.6 ± 77.5	231.2 ± 106.2	0.339
Fasting insulin, uIU/mL	32.4 ± 42.8	53.6 ± 78.7	0.668
CGM metrics			
Average glucose, mg/dL	154.6 ± 42.7	188.8 ± 35.6	0.021
GMI, %	7.0 ± 1.5	8.2 ± 1.3	0.024
CV, %	27.6 ± 9.6	30.2 ± 7.1	0.339
TAR, %	25.5 ± 26.2	44.6 ± 22.8	0.099
TIR, %	73.2 ± 26.0	55.1 ± 22.8	0.099
TBR, %	1.3 ± 2.6	0.3 ± 0.5	0.466
GMI-HbA1c, %	0.0 ± 1.6	2.0 ± 1.3	<0.001
Treatment			
Ventilator care, n (%)	25 (89.3)	8 (100.0)	1
Steroid, n (%)	21 (75.0)	6 (75.0)	1
Steroid dose, mg/day*	25.6 ± 24.8	61.7 ± 25.8	0.007
Insulin, n (%)	17 (60.7)	7 (87.5)	0.224
Insulin dose, IU/day	50.8 ± 61.2	71.7 ± 62.2	0.383

Converted to methylprednisolone.

APACHE, Acute Physiology and Chronic Health Evaluation; ARDS, acute respiratory distress syndrome; CGM, continuous glucose monitoring; CV, coefficient of variation; DM, diabetes mellitus; GMI, glucose management indicator; HbA1c, glycated hemoglobin; HDL, high-density lipoprotein; ICU, intensive care unit; TAR, time above range; TBR, time below range; TIR, time in range.

### The Effect of CGM Metrics on Clinical Outcomes

The effects of HbA1c, fasting glucose, and CGM metrics on the duration of ICU stay and 28-day mortality rate were analyzed using linear regression analysis and Cox regression analysis, respectively. Age, sex, presence of DM, APACHE score, and dose of steroid administered were considered as covariates.

Before adjustments, HbA1c, average glucose level, GMI, TAR, TIR, and GMI-HbA1c were significant risk factors for ICU stay (all p<0.05). After adjustments for covariates, GMI-HbA1c (adjusted coefficient = 2.34; 95% CI 0.54–4.14; p=0.017) remained as an independent risk factor for ICU stay (**Table 4**).

**Table 4:** Effect of CGM metrics on ICU stay.

	Crude coeff. (95%CI)	CrudeP value	Adjusted coeff. (95%CI)	adjusted P value
HbA1c	0.03 (0, 0.06)	0.049	-0.79 (-2.9, 1.31)	0.466
Fasting glucose	-0.36 (-1.81, 1.09)	0.63	0.03 (-0.01, 0.07)	0.11
Average glucose	0.06 (0.01,0.12)	0.04	0.06 (-0.01, 0.13)	0.089
GMI	1.76 (0.18,3.34)	0.036	1.84 (-0.16, 3.85)	0.082
CV	0.04 (-0.24, 0.32)	0.774	-0.04 (-0.39, 0.31)	0.83
TAR	0.10 (0.01, 0.19)	0.034	0.11 (-0.01, 0.22)	0.071
TIR	-0.10 (-0.19, -0.01)	0.041	-0.1 (-0.22, 0.01)	0.086
TBR	-0.64 (-1.72, 0.44)	0.255	-0.67 (-1.87, 0.52)	0.664
GMI-HbA1c	1.68 (0.33, 3.02)	0.02	2.34 (0.54, 4.14)	0.017

Linear regression analysis was performed. In the adjusted model, age, sex, presence of DM, APACHE score, and steroid dose (mg/day) were adjusted for each metrics.

CGM, continuous glucose monitoring; CI, confidence interval; CV, coefficient of variation; DM, diabetes mellitus; GMI, glucose management indicator; HbA1c, glycated hemoglobin; TAR, time above range; TBR, time below range; TIR, time in range.

In the aspect of 28-day mortality, before adjustments, GMI-HbA1c was an only significant risk factor (p=0.003). After adjustments for covariates, HbA1c (adjusted HR=0.13; 95% CI 0.02-0.99; p=0.049) and GMI-HbA1c (adjusted HR=2.42; 95% CI 1.01–5.76; p=0.046) were significant risk factors for 28-day mortality rate (**Table 5**).

**Table 5:** Effect of CGM metrics on 28-day mortality rate.

	Crude HR (95%CI)	CrudeP value	Adjusted HR (95%CI)	adjusted P value
HbA1c	0.65 (0.31, 1.34)	0.245	0.13 (0.02, 0.99)	0.049
Fasting glucose	1.01 (1, 1.01)	0.13	1 (0.99, 1.01)	0.922
Average glucose	1.01 (1.00,1.03)	0.052	1.01 (0.99, 1.03)	0.451
GMI	1.46 (1.00,2.15)	0.053	1.25 (0.7, 2.24)	0.453
CV	1.03 (0.96,1.10)	0.430	1.02 (0.93, 1.13)	0.656
TAR	1.02 (1.00,1.05)	0.076	1.01 (0.98, 1.05)	0.386
TIR	0.98 (0.95,1.00)	0.088	0.99 (0.95, 1.02)	0.429
TBR	0.69 (0.30,1.54)	0.363	0.67 (0.25, 1.77)	0.418
GMI-HbA1c	1.92 (1.25,2.96)	0.003	2.42 (1.01, 5.76)	0.046

A Cox regression analysis was performed. In the adjusted model, age, sex, presence of DM, APACHE score, and steroid dose (mg/day) were adjusted for each metrics.

CGM, continuous glucose monitoring; CV, coefficient of variation; DM, diabetes mellitus; GMI, glucose management indicator; Hba1c, glycated hemoglobin; HDL, high-density lipoprotein; HR, hazard ratio; TAR, time above range; TBR, time below range; TIR, time in range.

## Discussion

We demonstrated that poor CGM metrics were associated with longer ICU stay and higher 28-day mortality rate. In particular, GMI-HbA1c, which indicates the acute glycemic gap (8), was associated with prolonged ICU stay and was higher in non-survivors than in survivors in both patients without DM (0.6 vs. 2.3%, p=0.025) and with DM (-0.7 vs. 1.6, p=0.008). For every 1% increase in GMI-HbA1c, the duration of ICU stay was prolonged by 2.3 times and the 28-day mortality rate was increased 2.4 times, irrespective of age, sex, presence of DM, APACHE score, and dose of steroid administered.

Traditional POC glucose measurements are considered to be accurate and reliable and have the advantage of providing quick results compared to central laboratory measurements (28). CGM in critically ill patients is not only as effective as POC, but also reduces hypoglycemic events (29, 30) and nursing workload, and is cost effective (31, 32). It may be argued that placing a subcutaneous CGM can be disadvantageous in some clinical scenarios that may occur in the ICU setting, such as hypoperfusion. In fact, an intravascular microdialysis CGM showed superior accuracy compared to the subcutaneous CGM in cardiac surgery (33). Recently, however, the results of subcutaneous CGM are reported to be consistent irrespective of the use of vasopressors, mechanical ventilation, high-dose glucocorticoids, renal replacement therapy, and anasarca and even after surgery (34, 35). In respect of accuracy, the mean absolute relative difference (MARD) in this study was 15.5% (data not shown), which was higher than the recommended cut-off (9%) in general population, but consistent to the previously reported MARDs in ICU setting: 13.9% in Dexcom G6 (Dexcom, San Diego, USA) (36), 7.0% to 30.5% in FreeStyle Navigator or FreeStyle Libre (Abbott Diabetes, Alameda, USA) (37), and 14.0% to 23.7% in Guardian REAL-Time (Medtronic, Californea, USA) (37). In April 2020, the US FDA exercised enforcement discretion for the temporary use of inpatient CGM during the pandemic, and a recent report suggested an acceptable accuracy of CGM in critical care setting (36). Therefore, CGM can be an accurate, reliable, and practical method for glucose monitoring in an ICU setting (38–40).

A recent study using CGM technology concluded that 10–14 days of CGM data provide a good estimate of CGM metrics for a 3-month period (41). In this study, we only attached CGM for 5.5 ± 0.8 days, which was insufficient to determine long term glycemic control. However, we presented a new indicator, GMI-HbA1c, and its potential as a key clinical prognostic factor in acutely ill phase. Critically ill patients admitted to the medical ICU had high levels of inflammatory markers; accordingly, their blood glucose levels were also high. In addition, the use of high-dose steroid might have induced acute glycemic gap. Even after adjusting for these confounders, our results suggested that favorable outcomes can be achieved by reducing acute glycemic gap derived from GMI-HbA1c. GMI is an estimated A1c, which is calculated from a formula derived from the regression line computed from a plot of mean glucose concentration points on the x-axis and contemporaneously measured A1C values on the y-axis (8). Indeed, 22% of subjects showed discordance between GMI and HbA1c of >1% (3). Contrary to our expectations, there was no difference in HbA1c between survivors and non-survivors (7.0 ± 1.9 vs. 6.2 ± 0.5, p>0.05). Rather, the GMI-HbA1c was revealed to be a more reliable predictor for 28-day mortality. Therefore, understanding the differences between CGM-derived GMI and laboratory HbA1c may aid in safe and effective clinical management (42). GMI-HbA1c is easy to calculate, can assess acute or dramatic changes in blood glucose levels, and can be used as an index for personalized glucose management (8). Stringent glucose control is required if GMI is higher than HbA1c, to minimize excessive hyperglycemia. Conversely, if GMI is lower than HbA1c, less stringent glucose control may be needed to avoid hypoglycemic events (43). One thing to note is that the GMI-HbA1c should be interpretated considering various physical and biological factors. The GMI formula was derived from a cohort of adult patients mainly affected by Type 1 diabetes (8), and the hemoglobin glycosylation and red blood cell survival alter in the critically ill phase. Therefore, further clinical studies assessing GMI-HbA1c in various patient groups might reveal the effect of acute hypo- or hyperglycemic gaps on clinical outcomes.

We demonstrated that acute hyperglycemia and larger glycemic gap reflected by CGM metrices increased ICU stay and 28-day mortality rate in patients with and without DM. Newly diagnosed hyperglycemia affects in-hospital mortality and functional outcomes, regardless of a history of DM (44). In a study of patients with DM who underwent ICU care, the glycemic gap (mean blood glucose level during the first 7 days after admission to ICU minus the HbA1c-derived average blood glucose level) was an independent risk factor for 28-day mortality rate (27). Another study of patient without DM who underwent percutaneous coronary intervention, glycemic variability, based on the MAGE, increased the risk of 3-month major adverse cardiovascular events and mortality (45). Taken all, glucose monitoring using CGM metrics, and its appropriate management are required for critically ill patients, even those without DM.

The main strength of this study is that it documents the effect of the acute glycemic gap (GMI-HbA1c) on the risk of ICU stay and 28-day mortality rate, which has been less explored. In addition, this study showed the clinical implications of CGM in non-diabetic patients in the ICU setting. Despite these strengths, this study had several limitations. First, the number of patients was relatively small, and the patients enrolled were limited to those with medical conditions (especially respiratory disease); thus, selection bias may exist. Second, the recruited patients were infected, and hypoglycemic events did not occur; the TBR of all patients was approximately 1%. Third, since GMI is meant to represent the recent 10-14 days average glucose levels, it is required for the acquisition of CGM data for at least 10 days. However, we wanted to employ early phase ‘GMI’ within the first 3-days following admission to provide additional information for acutely ill patients - even if this did not mean the ‘average glucose’ indicator for a couple of weeks, as it intends to be used. Previous studies also consistently demonstrated the usefulness of the first 3- 5 days CGMS metrics in acute-ill patients (13, 29, 31). Further large and prospective studies using CGMS are warranted whether tight glycemic control is beneficial or not, or novel metrics for predicting mortality in medical or surgical ICU settings.

In conclusion, the acute glycemic gap (GMI-HbA1c) increased the risk of ICU stay and 28-day mortality rate irrespective of the presence of DM. CGM of critically ill patients in ICU settings is useful, and CGM metrics need to be studied in more detail.

## Data Availability Statement

The raw data supporting the conclusions of this article will be made available by the authors, without undue reservation.

## Author Contributions

JSM conceived the idea. SMC, EYC, and JSM designed the study. EYH, IRP, and YYL collected the data. EYH and SMC analyzed and interpreted the data and drafted the manuscript. JSM critically revised the manuscript. All authors gave final approval and agreed to be accountable for all aspects of the work, ensuring integrity and accuracy.

## Funding

This study was supported by a research grant funded by the Korean Diabetes Association (2019F-5 to SMC) and by a National Research Foundation of Korea grant funded by the Korean government (grant no. NRF-2019M3E5D1A02068242 to JSM).

## Conflict of Interest

The authors declare that the research was conducted in the absence of any commercial or financial relationships that could be construed as a potential conflict of interest.

## Publisher’s Note

All claims expressed in this article are solely those of the authors and do not necessarily represent those of their affiliated organizations, or those of the publisher, the editors and the reviewers. Any product that may be evaluated in this article, or claim that may be made by its manufacturer, is not guaranteed or endorsed by the publisher.
