# Supplementary material for: Novel Glycemic Index Based on Continuous Glucose Monitoring to Predict Poor Clinical Outcomes in Critically Ill Patients: A Pilot Study
Source: Front Endocrinol (Lausanne). 2022 May 4;13:869451. doi: 10.3389/fendo.2022.869451 (PMC9114696; doi:10.3389/fendo.2022.869451)
Supplement: Supplementary file 1 [file Table_1.docx]

Supplementary Material

**Supplementary Table 1.** **Association between classic variability indices and ICU stay and 28-day mortality**

|  | **ICU stay** | | **28-day mortality** | | |
| --- | --- | --- | --- | --- | --- |
|  | Correlation coefficients | p-value | Survivor | Non-survivor | p-value |
| MAGE | 0.141 | 0.443 | 4.4 ± 1.9 | 5.1 ± 2.2 | 0.284 |
| CONGA | 0.327 | 0.055 | 7.8 ± 2.0 | 10.0 ± 2.0 | 0.015 |
| M-value | 0.320 | 0.061 | 8.2 ± 9.2 | 17.5 ± 13.3 | 0.059 |

MAGE, mean amplitude of glucose excursion; CONGA, continuous overall net glycemic action
